# Can we afford to treat obstructive sleep apnoea?

**DOI:** 10.7196/AJTCCM.2020.v26i3.067

**Published:** 2020-09-16

**Authors:** Richard Raine

**Affiliations:** Department of Pulmonology, Division of Medicine, University of Cape Town and Groote Schuur Hospital, Cape Town, South Africa

**Keywords:** Obstructive sleep apnoea, CPAP, Cardiovascular disease

## Editorial

Obstructive sleep apnoea (OSA) is a common and ubiquitous problem, and this journal has recently published articles from Nigeria and Burkina Faso to demonstrate awareness of the syndrome in West Africa.^[1,2]^ The prevalence of OSA in many countries is not known, although the figures of 4% of men and 2% of women identified as having OSA in the Wisconsin Sleep Cohort^[3]^ are likely to be an underestimate given the increase in obesity around the world. Recently, the global prevalence of OSA was estimated using known prevalence data (where available), population body mass index and racial demographics.^[4]^ Results from this analysis suggested that in South Africa (SA), 22.8% of the adult population has OSA, with an apnoea-hypopnoea index (AHI) >15/hour. Estimates for Burkina Faso and Nigeria were 5.4% and 22.8%, respectively. This means that there is a huge burden of patients with sleep-disordered breathing with neurocognitive dysfunction, decreased quality of life, cardiovascular disease and increased risk of death who need to be identified and treated.

The article published by Ouédraogo *et al*. ^[5]^ (from Burkina Faso) in this issue of the *AJTCCM* describes the clinical features, diagnosis and proposed management of 77 patients presenting to their pulmonology department with symptoms suggestive of OSA. Continuous positive airway pressure (CPAP) therapy was recommended for 41 patients, of whom only 9 could purchase the device. An orthodontic appliance/mandibular advancement device (MAD) was recommended for 27 patients, none of whom could obtain one. Weight loss with lifestyle and dietary modifications was the only treatment available to most of the patients.

This highlights the greatest problem in the management of OSA – the best treatment is unaffordable for most of the population. Available effective treatment modalities for OSA include weight loss, MAD and CPAP. Surgery and upper airway stimulation may be useful in a very small and highly selected group of patients, and both have significant associated morbidity.

Weight loss is potentially available to all. Lifestyle and dietary modifications are very useful interventions to reduce associated comorbidities. Weight loss itself, whether via medical measures or bariatric surgery, is only modestly effective in reducing AHI and improving sleepiness scores.^[6]^ MAD treatment is effective in reducing snoring, but has a variable effect on sleepiness. These devices are usually more effective in individuals with more normal BMI and lower AHI.^[7]^ MAD therapy costs ~ZAR4 000 in SA, but considerable expertise is required for fitting and customisation, and ‘titration’ to assess effectiveness prior to fitting is not possible.

CPAP remains the most effective treatment for OSA, with large improvements in machine technology and mask construction since the first description of CPAP use by Sullivan *et al*. ^[8]^ in 1981. Good short-term improvements in quality of life and sleepiness have been reported,^[9,10]^ but two large studies of CPAP use in patients with incident cardiovascular disease, but not excessive sleepiness, have failed to alter the cardiovascular outcomes.^[11,12]^ Of note, however, in these studies mean compliance with CPAP use was 3.3 and 2.8 hours, respectively. A Finnish study has shown better event-free survival with better compliance of 6.4 hours per night.^[13]^ Longer-term use for more than 6 years has shown improved survival in patients enrolled in the Sleep Heart Health Study.^[14]^

These results appear congruent with symptom subtypes described from the Sleep Heart Health Study, where four subtypes have been described: disturbed sleep; minimally symptomatic; excessively sleepy; and moderately sleepy. The excessively sleepy subtype had significantly increased risk for incident cardiovascular disease, coronary heart disease and heart failure.^[15]^ This suggests that the patients most likely to benefit from CPAP use are those in the excessively sleepy subtype

Unfortunately CPAP remains an expensive therapy, and is not accessible to many in sub-Saharan Africa. The reason for failure to use CPAP in most patients in Burkina Faso was cost (machines imported from Europe cost between EUR900 and 2 000).^[5]^ In SA, the presence of companies acting as agents for CPAP machines means that prices are lower, with reasonable quality fixed-level CPAP machines costing from ZAR5 000 upwards, with a mask and headgear adding ZAR1 500 - 2 500. Even this is too high a price for many of our patients, and attempts to access machines through the state have been mostly unrewarding. A group in Barcelona has been working on producing off-the-shelf, low-cost technological solutions for low-income countries, and has produced schematics for a fixed level CPAP machine using components bought using e-commerce and 3D printing. The estimated cost for this device was EUR60.^[16]^

The message for CPAP use is to use it for as many hours as possible per night and for a long period, before cardiovascular benefit is seen. Excessively sleepy patients should be prioritised for treatment. Inventive solutions for producing lower-cost machines may be one of the ways to manage this disease, with increasing impact on health and quality of life.
